# Epidemiology of chronic kidney disease in a Sri Lankan population

**DOI:** 10.4103/0973-3930.43101

**Published:** 2008

**Authors:** I. K. Gooneratne, A. K. P. Ranaweera, N. P. Liyanarachchi, N. Gunawardane, R. D. Lanerolle

**Affiliations:** Department of Clinical Medicine, Faculty of Medicine, University of Colombo, Sri Lanka; 1 Department of Community Medicine, Faculty of Medicine, University of Colombo, Sri Lanka

**Keywords:** Chronic kidney disease, diabetic nephropathy, epidemiology in Sri Lanka

## Abstract

**CONTEXT:**

Chronic kidney disease (CKD) is characterized by progressive destruction of renal mass with irreversible sclerosis and loss of nephrons over a period of months to years, depending on the underlying etiology.

**AIM:**

To describe demographic patterns and identify common causes of CKD in patients admitted to ward 41 and 48B, National Hospital of Sri Lanka.

**SETTINGS AND DESIGN:**

A hospital based descriptive 3-month study was conducted at ward 41 and 48B, National Hospital of Sri Lanka. A case record form was used to record sociodemographic variables, stage of renal disease, and etiology of patients in established chronic renal failure. Sources of data included patient interviews, diagnosis cards and case records, ultrasound scan reports, and biopsy findings.

**RESULTS:**

One hundred and twenty-one patients were recruited with male to female ratio being 2.5:1 (86:35). Mean age of the population was 47.8 years (SD ± 13.7). Common causes of CKD identified in these patients included diabetic nephropathy (37, 30.6%), hypertension (16, 13.2%), glomerulonephritis (12, 9.9%), and obstructive uropathy (10, 8.3%). The cause was unknown in 25.6% of patients with chronic renal disease. Fifty percent of patients were from the Western Province. The leading cause of CKD in patients from the Western Province was diabetic nephropathy (26, 37.7%). The etiology of CKD was unknown in majority of the patients (14, 27.4%) from other provinces. The difference in incidence of diabetic nephropathy in the Western Province as to other provinces was not statistically significant (*P* > 0.05).

**CONCLUSION:**

Diabetes is a major contributor to CKD reflecting changing disease epidemiology in Sri Lanka.

## Introduction

Chronic kidney disease (CKD) is characterized by progressive destruction of renal mass with irreversible sclerosis and loss of nephrons over a period of months to years, depending on the underlying etiology.[1] CKD is defined as either kidney damage or decreased kidney function (decreased GFR) for three or more months.[2] Chronic renal failure (CRF) requiring dialysis or transplantation is known as end-stage renal disease (ESRD).[3]

Incidence rates, etiology, and demographic patterns in Sri Lanka are largely unknown and only a few studies have been published. In one such study by Ramchandran, observations were made in 1387 patients having renal disease during a 5-year period from 1989–1993 at the General Hospital of Sri Lanka.[4] These patients were classified as renal disease presenting with acute renal failure (448), CKD (336) and renal disease not resulting in renal failure (603). Out of the 336 patients with CKD, 28% had ESRD of unknown origin with one-thirds of the patients having small kidneys and 22% having chronic glomerulonephritis. Nonobstructive pyelonephritis was observed in 14% of the patients. Other important causes for CKD were diabetic nephropathy (12%), obstructive uropathy (13%) and polycystic disease (2%). The mean age of patients with CKD due to pyelonephritis, glomerulonephritis, and ESRD due to unknown causes was 34 years. The mean ages for patients with diabetic nephropathy, obstructive uropathy, and polycystic disease were 50, 40, and 46 years, respectively.

Since data on etiology and demographic patterns are limited, further studies in the Sri Lankan population are justified. This study describes demographic patterns and identifies common causes of CKD in patients admitted to ward 41 and 48B, which functions as the nephrology unit of the National Hospital of Sri Lanka.

## Materials and Methods

A descriptive hospital-based 3-month study was conducted at ward 41 and 48B of the National Hospital of Sri Lanka in 2006. A case record form was used to record sociodemographic variables, stage of renal disease, and probable etiology of patients already in established CKD. The sources of data included patient interviews, diagnosis cards and case records, ultrasound scan reports, and biopsy findings.

The probable causes of chronic renal disease were made according to the following criteria.

A diagnosis of diabetic nephropathy was made with the presence of clinically and bio-chemically confirmed diabetes mellitus and one of the following criteria: long duration of diabetes before the onset of CKD (minimum of 10 years), normal sized kidneys by ultrasound, or presence of diabetic retinopathy by fundus examination.[5]

As renal biopsy and morphological examination is rarely performed,[6] a diagnosis of CKD due to hypertension was made with the following criteria: long-standing hypertension (minimum of 5 years), hypertension preceding renal dysfunction, and no evidence of any other renal disease.[7]

The other causes of CKD were established on biopsy and ultrasound scan report findings.

The stage of chronic renal disease was established by recording the most recent (within the last 3 months) 24 hour urinary creatinine clearance. Staging was done according to the Kidney Disease Outcomes Quality Initiative (K/DOQI) guidelines.[8]

The place of residence for at least the last ten years of each individual was recorded.

Statistical analysis was done by SPSS version 11. Informed written consent was obtained from every individual belonging to the study population. Ethical approval was obtained from ethical review committees of the National Hospital of Sri Lanka and Faculty of Medicine, University of Colombo.

## Results

One hundred and twenty-one patients were recruited with male to female ratio being 2.5:1 (86:35). Majority of patients were Sinhalese (91, 75.2%). Tamil (15, 12.4%) and Muslim (15, 12.4%) patients made up the minority. The mean age of the population was 47.8 years with the minimum age being 20 and the maximum being 83. Most of the patients were from the Western Province (57%). Others were from: Central (1.7%), Eastern (0.8%), North Central (1.7%), North Western (14.9%), Northern (5.0%), Sabaragamuwa (5.8%), Southern (9.9%), and Uva (2.5%).

Common causes of CKD identified in these patients included diabetic nephropathy (37, 30.6%), hypertension (16, 13.2%), and glomerulonephritis (12, 9.9%). The cause was unknown in a significant percentage of patients with chronic renal disease (31, 25.6%) [Table 1].

**Table 1:** Etiology of chronic renal disease

Etiology	Number of subjects	Percentage
Diabetic nephropathy	37	30.6
Hypertension	16	13.2
Interstitial disease	3	2.5
Glomerulonephritis	12	9.9
Obstructive uropathy	10	8.3
Adult polycystic kidney disease	7	5.8
Unknown	31	25.6
Others	5	4.1
Total	121	100

The stage of CKD was unknown in 25 patients (20.7%). The majority of patients were in end-stage renal failure at presentation (66.9%). Stage 3 and 4 constituted a minority (3.3% and 4.1%, respectively). In 5% of the patients, a diagnosis of acute on CKD was made [Table 2].

**Table 2:** Stage of chronic renal disease

Stage	Frequency	Percentage
3	4	3.3
4	5	4.1
5	81	66.9
Acute on chronic	6	5.0
unknown	25	20.7
Total	121	100

The leading cause of CKD in patients from the Western Province was diabetic nephropathy (26, 37.7%). However, in the majority of patients from other provinces the etiology of CKD was unknown (14, 27.4%). This number was distributed among the following provinces: Eastern (1), North Western (4), Northern (2), Sabaragamuwa (2), Southern (3), and Uva (2) [Table 3].

**Table 3:** Comparison of etiology between Western and other provinces

Etiology provinces n (%)	Western province n (%)	Other
Diabetic nephropathy*	26 (37.7)	11 (21.6)
Hypertension	8 (11.6)	8 (15.7)
Glomerulonephritis	6 (8.7)	6 (11.7)
Unknown	17 (24.6)	14 (27.4)
Obstructive uropathy	4 (5.8)	6 (11.8)
Adult polycystic kidney disease	4 (5.8)	3 (5.9)
Interstitial disease	1 (1.4)	2 (3.9)
Others	3 (4.3)	1 (1.96)
Total	69 (100)	51(100)

* Diabetic nephropathy, χ = 3.5698, df = 1, *P* > 0.05

The difference in the incidence of diabetic nephropathy between Western Province and other provinces was not statistically significant (*P* > 0.05).

A total number of 58 patients were below the age of 50 years. The common causes of CKD in this age group included diabetic nephropathy (19%), glomerulonephritis (15.5%), and hypertension (10.3%). The majority of patients had CKD of unknown cause (44.8%).

In 63 patients who were ≥50 years, the following causes of CKD were identified: diabetic nephropathy (41.3%), hypertension (15.9%), obstructive uropathy (12.8%), and adult polycystic kidney disease (7.9%). Only 7.9% of patients in this age category had CKD of unknown etiology [Table 4].

**Table 4:** Comparison of etiology between age categories

Etiology	Age <50 years n (%)	Age ≥ 50 years n (%)
Diabetic nephropathy*	11 (19)	26 (41.3)
Hypertension	6 (10.3)	10 (15.9)
Interstitial disease	1 (1.7)	2 (3.2)
Glomerulonephritis	9 (15.5)	3 (4.8)
Obstructive uropathy	2 (3.4)	8 (12.8)
Adult polycystic kidney disease	2 (3.4)	5 (7.9)
Unknown**	26 (44.8)	5 (7.9)
Others	1 (1.7)	4 (6.4)
Total	58 (100)	63 (100)

* Diabetic nephropathy, χ = 7.077, df = 1, *P* < 0.01

** Unknown, χ = 21.567, df = 1, *P* < 0.001

The difference in the incidence of CKD due to diabetic nephropathy between the two age categories is statistically significant (*P* < 0.01). The difference in the incidence of CKD due to unknown etiology between the two age categories is statistically significant (*P* < 0.001).

Common causes of CKD in patients from the Western Province who were ≥50 years are as follows: diabetic nephropathy (47.2%), hypertension (11.1%), obstructive uropathy (11.1%), and adult polycystic kidney disease (8.3%). 8.3% of patients in this age category had CKD of unknown etiology. The leading causes of CKD in other provinces include: diabetic nephropathy (34.6%), hypertension (23.1%), and obstructive uropathy (15.4%). The difference in the incidence of diabetic nephropathy in the Western Province as to other provinces was not statistically significant (*P* > 0.05) [Table 5].

**Table 5:** Comparison of etiology between Western and other provinces in the ≥50 years category

Etiology	Western province n (%)	Other provinces n (%)
Diabetic nephropathy*	17 (47.2)	9 (34.6)
Hypertension	4 (11.1)	6 (23.1)
Interstitial disease	1 (2.7)	1 (3.8)
Glomerulonephritis	2 (5.6)	1 (3.8)
Obstructive uropathy	4 (11.1)	4 (15.4)
Adult polycystic kidney disease	3 (8.3)	2 (7.7)
Unknown	3 (8.3)	2 (7.7)
Others	2 (5.6)	1 (3.8)
Total	36 (100)	26 (100)

* Diabetic nephropathy, χ 0.985, df = 1, *P* < 0.01

## Discussion

The attention paid globally to CKD is attributable to five factors: rapid increase in its prevalence, the enormous cost of treatment, recent data indicating that overt disease is the tip of an iceberg of covert disease, an appreciation of its major role in increasing the risk of cardiovascular disease, and the discovery of effective measures to prevent its progression. These factors render CKD an important focus of healthcare planning.[9]

Chronic glomerulonephritis and interstitial nephritis are the leading causes of CKD in the developing world reflecting the high rates of infection.[9] In a study done by Ramachandran in the early 1990s at the same institution as in our study, the leading causes of CKD were chronic glomerulonephritis and interstitial nephritis reflecting the trend in developing countries.[4] However, the leading causes of CKD in our patient population were diabetic nephropathy and hypertension. Though these results cannot be generalized to the Sri Lankan population as it is representative of institutional data, change in trends of CKD in Sri Lanka is suggested by this study. The results in our study are comparable with trends in western countries such as the United States. During 1990–2001, the prevalence of chronic kidney failure in the United States increased to 104%, from 697 to 1,424 cases/million population; with the largest increase being in the prevalence of diabetes-related chronic kidney failure (increased to 194%, from 171 to 503 cases/million population). The prevalence of hypertension-related chronic kidney failure increased to 99% (from 166 to 331 cases/million population). These were the two leading causes of CKD in the United States in 2001 with diabetes being the leading contributor.[10]

The prevalence of diabetes has reached epidemic proportions. World Health Organization predicts that developing countries will bear the brunt of this epidemic in the 21st century, with approximately 80% of all new cases of diabetes expected to appear in the developing countries.[11] The Sri Lankan situation is similar with an estimated 2.8 million diabetics. The increase in diabetes can be attributed to urbanization, lifestyle changes, and aging.[12] The Western Province consists of an urbanized population. The majority of patients in our study were from the Western province (57%), which may explain the high proportion of diabetic nephropathy. This result may further be strengthened by a greater proportion of diabetic nephropathy seen in patients from the Western Province (37%) as compared to patients from other provinces (21%). However, this difference was not statistically significant possibly due to the small study population. The aforesaid result supported by the fact that a significant proportion of diabetics with CKD came from other provinces, seems to suggest a greater contribution of diabetes toward CKD in the rural setting as well. Diabetes may have a greater impact on the incidence of CKD in Sri Lanka, contrary to what was previously thought.

The majority of the CKD admissions to the professorial unit consisted of patients with ESRD (66.9%) reflecting late referral and diagnosis. There is considerable evidence that complications of diabetes such as cardiovascular and renal disease are common among South Asians.[13] The major contributor toward ESRD in this population was diabetes (28.4%) and CKD of unknown etiology (27%). In a previous study at the same institution, the main contributor toward ESRD was CKD of unknown causes (28%).[4] Thus, the above result brings out the changing trends in CKD.

CKD of unknown etiology has been a major contributor toward ESRD in previous studies. Fluorosis and aluminum toxicity causing interstitial disease were postulated as possible causes of CKD in this group of patients in the past.[14] This fact was mainly highlighted in patients originating from the North Central Province. Though the main contributor of CKD in patients from “other provinces” was CKD of unknown cause, there were no patients from the North Central province, which may be due to geographical selection bias. The main contributor toward CKD in the younger age group (<50 years) in our study has been CKD of unknown etiology (44.8%). The difference in the incidence of CKD due to unknown etiology between patients aged <50 years and patients aged ≥50 years was statistically significant. CKD of unknown etiology seems to be more prevalent in a younger age group. This result may be explained by environmental factors and by missed childhood infections.

The major contributors toward CKD in the older age group (≥50 years) were diabetes (26, 41.3%) and hypertension (10, 15.9%). The difference in the incidence of diabetic nephropathy between the older and younger age groups was statistically significant. Since diabetes and hypertension are diseases that become more prevalent with increasing age[15,16] the above result is to be expected.

In conclusion, diabetes is a major contributor toward chronic renal disease and ESRD reflecting changing disease demographics in Sri Lanka.
